# 成人原发性血小板增多症诊断与治疗中国指南（2026年版）

**DOI:** 10.3760/cma.j.cn121090-20260212-00089

**Published:** 2026-05

**Authors:** 中华医学会血液学分会血栓与止血学组

## Abstract

原发性血小板增多症（essential thrombocythemia，ET）是一类以骨髓巨核细胞过度增殖及外周血血小板计数持续增高为特征的经典型费城染色体阴性骨髓增殖性肿瘤，主要临床风险为血栓与出血事件，少数患者存在进展为骨髓纤维化或急性白血病的风险。近年来，随着分子检测、骨髓病理诊断、新药研发及临床管理水平的提升，ET规范化诊疗的需求日益突出。为向我国血液科医师提供可操作的临床实践指导，中华医学会血液学分会血栓与止血学组组织国内相关专家，基于国内外循证医学证据并结合专家共识，制订了本指南。

## Abstract (en)

Essential thrombocythemia（ET）is a classic Philadelphia chromosome-negative myeloproliferative neoplasm characterized by excessive proliferation of bone marrow megakaryocytes and sustained elevation of peripheral blood platelet counts. The primary clinical risks include thrombotic and bleeding events, with a minority of patients experiencing the risk of progression to myelofibrosis or transformation to acute leukemia. In recent years, the demand for standardized diagnosis and treatment of ET has become increasingly prominent with advances in molecular testing, bone marrow pathology diagnosis, novel drug development, and clinical management. To provide actionable clinical practice guidance for hematologists in China, the Thrombosis and Hemostasis Group of the Chinese Society of Hematology, Chinese Medical Association, convened domestic experts to formulate this guideline, based on both international and domestic evidence-based medical evidence combined with expert consensus.

原发性血小板增多症（essential thrombocythemia，ET）是起源于骨髓造血干/祖细胞的克隆性疾病，是费城染色体阴性的经典型骨髓增殖性肿瘤（myeloproliferative neoplasms，MPN）中常见的亚型之一[1]–[3]，其年发病率在欧美国家为（0.38～1.94）/10万，在东亚地区为（0.77～1.07）/10万（包括年龄标准化后的发病率）[4]–[6]。疾病特征为骨髓巨核细胞过度增殖且伴有形态异常，外周血PLT持续增高，部分患者伴有血栓或出血并发症，少数患者存在进展为骨髓纤维化或急性白血病的风险[1]–[2],[7]。近年来，关于成人ET的诊断标准、预后分层与治疗策略均有显著进展。为向我国血液科医师提供规范化的临床实践指导，中华医学会血液学分会血栓与止血学组组织国内相关专家，参考美国国家综合癌症网络（National Comprehensive Cancer Network，NCCN）肿瘤学临床实践指南[8]、中国临床肿瘤学会（Chinese Society of Clinical Oncology，CSCO）恶性血液病诊疗指南[9]、亚洲髓系工作组（Asian Myeloid Working Group，AMWG）关于经典型费城染色体阴性MPN管理的专家共识[10]及欧洲白血病网（European LeukemiaNet，ELN）专家组推荐意见[11]，结合我国临床实践特点，制订了本指南。

一、指南制订方法

1. 指南发起与制订过程：本指南由中华医学会血液学分会血栓与止血学组发起并组织实施。指南制订工作于2025年5月启动，2026年2月定稿。

2. 指南工作组构成及分工：本指南工作组由来自全国多家医疗机构的血液学、检验学及病理学专家组成，同时邀请循证医学相关人员参与方法学支持。根据指南制订需要，工作组分为指导组、撰写组和审阅组。指导组负责总体设计及重要问题决策；撰写组负责证据整理、文本起草及修改；审阅组对指南内容进行审核并提出修改意见。各组成员在指南制订过程中分工协作，共同完成指南的制订。

3. 利益冲突管理：所有制订工作组成员均填写了利益冲突声明表，均声明不存在与本指南相关的经济或非经济利益冲突。制订过程或推荐意见形成未受到资助影响。

4. 指南注册：本指南已在国际实践指南注册与透明化平台（https://www.guidelines-registry.cn）注册（注册号：PREPARE-2025CN1276）。

5. 目标人群与使用者：本指南的目标人群为成人ET患者，主要使用者为从事血液系统疾病诊疗的临床医师、护理人员等医务工作者。

6. 临床问题的遴选与确定：本指南围绕ET的诊断程序、诊断标准、鉴别诊断、预后评估及治疗管理中的关键问题，基于前期文献调研及临床实践需求，初步形成临床问题清单。随后通过专家讨论方式对问题进行筛选与优化，重点评估其临床重要性及可操作性，并对问题表述进行修订。经3轮专家讨论，最终确定纳入本指南的14个临床问题，用于指导后续证据整合及推荐意见的形成。

7. 证据的检索与评价：对最终纳入的临床问题，参考人群、干预、比较和结局（population，intervention，comparison，outcome，PICO）原则检索PubMed、Web of Science、ClinicalTrials.gov、International Clinical Trial Registry Platform、Embase、中国知网（China National Knowledge Infrastructure，CNKI）、万方数据知识服务平台及维普数据库等中英文数据库，检索时间范围为建库至2025年10月，在指南制订过程中进行针对性补充检索，及时纳入最新发表的高质量文献。检索采用主题词与自由词结合的方式，以“essential thrombocythemia”、“myeloproliferative neoplasms”、“diagnosis”、“risk stratification”、“treatment”等为主要英文关键词，同时结合“原发性血小板增多症”、“骨髓增殖性肿瘤”、“诊断”、“危险分层”、“治疗”等中文关键词进行扩展检索。随机对照试验采用Cochrane偏倚风险评估工具进行评价，观察性研究采用纽卡斯尔-渥太华量表进行评价，系统评价及Meta分析采用系统评价偏倚风险评价工具（Assessing the Methodological Quality of Systematic Reviews 2，AMSTAR2）进行方法学质量评估。

8. 推荐意见的形成：针对诊断标准、预后分层及疗效评价标准，本指南采用Delphi法形成专家共识。推荐意见获得≥85％专家支持定义为“强一致”，75％～84％定义为“一致”，65％～74％定义为“未达成共识”。对于治疗及预防治疗相关问题，本指南采用推荐分级的评估、制订与评价（Grading of Recommendations Assessment, Development and Evaluation，GRADE）方法学进行循证评价[12]。依据研究设计及GRADE体系所定义的五类降级因素（偏倚风险、不一致性、不直接性、不精确性和发表偏倚）与三类升级因素（效应量大、剂量反应关系及所有偏倚可能使效果趋向保守），将证据质量划分为高、中、低或极低四级（表1）。推荐意见的强度结合证据质量、风险-获益平衡、患者价值观与偏好及资源消耗和可及性等因素综合判断，分为强推荐和条件推荐。强推荐表示多数患者在大多数情境下会选择该措施，临床医师通常应予以实施；条件推荐则提示不同患者可能作出不同选择，临床决策需充分结合患者的价值观、偏好及具体情境进行共同决策。此外，对于需经专家意见进一步确认的治疗及预防治疗相关推荐，本指南亦辅以Delphi法开展一致性评估，并以上述一致性阈值（≥85％：强一致；75％～84％：一致；65％～74％：未达成共识）确定最终推荐意见。对于诊断程序及鉴别诊断相关内容，因其主要为临床评估、检查路径及疾病识别要点的描述，属于流程性和说明性内容，不单独形成推荐意见。诊断标准、预后分层、疗效评价标准以及部分治疗或预防条目为专家共识性管理原则，仅报告一致性强度。

**表1:** 推荐分级的评估、制订与评价（GRADE）证据质量分级及依据

等级	含义	典型证据类型	评定逻辑
高	对效应估计非常有信心；真实效应极可能接近估计值	设计良好的随机对照试验	无明显降级因素
中	对效应估计有中等信心；真实效应可能接近估计值，但也可能存在明显差异	存在局限性的随机对照试验；或以观察性研究为主但因升级因素从低升至中	存在1～2个降级因素，或观察性研究存在升级因素
低	对效应估计的信心有限；真实效应可能与估计值存在较大差别	观察性研究（前瞻性队列、回顾性队列、病例对照、单臂前瞻性干预研究）	存在多个降级因素
极低	几乎没有信心；真实效应可能与估计值存在极大差别	质量较差的观察性研究、病例系列、病例报告、专家意见	存在多个严重降级因素

9. 指南的外审与批准：推荐意见达成共识后，由执笔专家完成指南初稿撰写，经全体专家组成员讨论并同意后，提交外审小组进行同行评审，并对外审意见进行相应修订与完善，最终提交指导委员会审议通过后确定指南终稿。

10. 发布、传播与更新：本指南拟通过学术期刊发表等形式进行发布，并在相关学术会议及继续教育活动中推广应用。随着新的循证医学证据不断积累，指南内容将根据需要适时更新。

二、临床问题与推荐意见

（一）诊断程序

1. 病史采集与体格检查：需核实患者年龄，询问有无微循环障碍症状（如头痛、肢端感觉异常等）及体质性症状（如疲劳、不明原因发热、盗汗、体重下降等）。询问是否存在可能导致反应性血小板增多的疾病或病史（如感染、炎症性疾病、慢性失血、肿瘤或手术史，尤其是脾切除或脾栓塞术）；是否存在可能导致假性血小板增多的疾病（如地中海贫血等）；是否使用可能引起血小板增多的药物（如类固醇激素、促血小板生成药物等）。同时，应评估心血管危险因素（cardiovascular risk factors，CVRF，包括高血压、高脂血症、糖尿病和吸烟等），并询问既往动脉或静脉血栓史、异常出血史及家族中是否存在类似病史。建议在初诊及随访过程中动态评估MPN总症状评估量表（Myeloproliferative Neoplasm Symptom Assessment Form Total Symptom Score，MPN-SAF TSS）。在初诊时及治疗过程中进行脾脏触诊，了解脾脏大小动态变化[8]–[9],[11],[13]–[16]。

2. 实验室检查：对于疑诊ET的患者，下列检查应作为必检项目：①外周血细胞计数及外周血涂片分类；②肝肾功能、电解质、血脂、乳酸脱氢酶、血清促红细胞生成素；③C反应蛋白（C reactive protein，CRP）、红细胞沉降率（erythrocyte sedimentation rate，ESR）、血清铁、转铁蛋白饱和度、总铁结合力及血清铁蛋白；④骨髓穿刺细胞学分析及骨髓铁染色；⑤骨髓活检病理细胞学分析及网状纤维（嗜银）染色；⑥JAK2 V617F、JAK2第12号外显子、CALR第9号外显子和MPL第10号外显子基因突变检测（骨髓或外周血），有条件时病程中动态监测基因突变负荷（每年1～2次）；⑦BCR::ABL1融合基因检测；⑧染色体核型分析；⑨流式细胞术免疫分析；⑩肝脏、脾脏影像学检查：肝脾彩色多普勒超声或腹部CT[1]–[2],[8]–[9],[13],[17]。

对于PLT明显升高（>1 000×10^9^/L）、出现不明原因出血表现或拟进行手术操作的患者，建议进行凝血酶原时间、活化部分凝血活酶时间、血管性血友病因子（von Willebrand factor，VWF）水平（包括VWF抗原及VWF活性）检测[18]。

有条件者推荐开展包括JAK2、CALR、MPL、LNK、CBL、ASXL1、TET2、DNMT3A、SRSF2、SF3B1、U2AF1和TP53等基因在内的靶向测序，用于辅助诊断及预后评估[8]–[9],[19]–[22]。

对于具有家族史，或血小板持续增多但无典型驱动基因（JAK2 V617F、CALR 9号外显子或MPL 10号外显子）突变的年轻患者，建议进行靶向测序或全外显子组测序，以筛查THPO、GSN、LNK、NFE2等突变及JAK2和MPL少见位点突变，排除遗传性或家族性血小板增多症[23]–[25]。

（二）诊断标准

**临床问题1**：疑似ET的患者应采用何种诊断标准进行确诊？

**推荐意见1**：推荐采用2022年国际共识分类（International Consensus Classification，ICC）标准进行ET的诊断（表2）（强一致）[1]。

**表2:** 2022年国际共识分类（ICC）原发性血小板增多症诊断标准

类别	诊断标准
主要标准	①血小板计数≥450×10^9^/L
	②骨髓活检可见巨核细胞增生程度显著增加，胞体大、核过分叶（鹿角状）的成熟巨核细胞数量增多，但较少成簇^a^；粒系、红系无显著增生或左移，无显著纤维化^b^
	③不能满足BCR::ABL1阳性慢性髓细胞性白血病、真性红细胞增多症、原发性骨髓纤维化及其他髓系肿瘤的诊断标准
	④存在JAK2、CALR或MPL基因突变^c^
次要标准	存在其他克隆性标志^d^或无反应性血小板增多的证据^e^

**注** 符合4条主要标准或前3条主要标准及次要标准。^a^≥3个巨核细胞紧密相邻为簇，原发性血小板增多症患者巨核细胞簇通常≤6个巨核细胞，大的巨核细胞簇（>6个巨核细胞）且伴有粒系增殖是纤维化前/早期原发性骨髓纤维化的形态学重要特征。^b^初诊时偶见骨髓纤维化分级1级者。^c^建议采用高敏感性方法检测JAK2 V617F（检测灵敏度<1％）、CALR和MPL（检测灵敏度1％～3％）突变；对于上述突变阴性病例，应进一步考虑筛查非典型JAK2和MPL基因突变。^d^通过细胞遗传学检查或采用高敏感度的二代测序技术进行评估。^e^反应性血小板增多可由多种潜在疾病引起，包括缺铁性贫血、慢性感染、慢性炎症性疾病、药物相关因素、恶性肿瘤及脾切除术后状态等

**临床问题2**：ET患者发生疑似疾病进展时，应采用何种标准诊断ET后骨髓纤维化（post-essential thrombocythemia myelofibrosis，post-ET MF）？

**推荐意见2**：推荐采用2022年ICC标准进行post-ET MF的诊断（表3）（强一致）[1]。

**表3:** 2022年国际共识分类（ICC）原发性血小板增多症（ET）后骨髓纤维化诊断标准

类别	诊断标准
主要标准	①此前确诊为ET
	②骨髓纤维化分级为2级或3级（按0～3级分级标准）
次要标准	①贫血：血红蛋白低于相应年龄、性别及海拔高度的参考范围，并较基线水平下降≥20 g/L
	②外周血出现幼稚粒细胞、幼稚红细胞
	③进行性脾大（可触及脾脏较基线增大≥5 cm，或出现新发可触及性脾大）
	④乳酸脱氢酶超过参考范围上限
	⑤以下3项体质性症状中至少具备2项：过去6个月内非意愿性体重下降>10％；夜间盗汗；不明原因发热（体温>37.5 °C）

**注** 诊断需满足2项主要标准和至少2项次要标准

**临床问题3**：ET患者疑似疾病进展至加速期或急变期时，应如何进行诊断界定？

**推荐意见3**：加速期定义为外周血或骨髓中原始细胞比例10％～19％，急变期定义为外周血或骨髓中原始细胞比例≥20％或出现髓系肉瘤（强一致）[8]。

（三）鉴别诊断

1. 反应性血小板增多症：常见原因包括感染、炎症性疾病、缺铁性贫血、过敏性疾病、恶性肿瘤、手术（尤其为脾切除或脾栓塞术）、酒精戒断及某些药物因素（如糖皮质激素、促血小板生成药物或化疗后的反弹性血小板增多）等[21]。感染或炎症性疾病常伴CRP或ESR升高。缺铁性贫血可通过血清铁、总铁结合力、转铁蛋白饱和度及血清铁蛋白等指标进行鉴别。

2. 其他可能导致血小板增多的血液系统克隆性疾病：包括真性红细胞增多症（polycythemia vera，PV）、原发性骨髓纤维化（primary myelofibrosis，PMF）、慢性髓细胞性白血病（chronic myelogenous leukemia，CML）、慢性粒-单核细胞白血病、骨髓增生异常肿瘤（myelodysplastic neoplasms，MDS）伴低原始细胞及孤立5q缺失、MDS/MPN伴SF3B1突变和血小板增多、MDS/MPN伴环形铁粒幼红细胞和血小板增多（非特指型，特征为SF3B1野生型且环形铁粒幼红细胞≥15％）、伴PDGFRB重排的髓系/淋系肿瘤、部分淋巴细胞增殖性疾病等[2],[13],[26]–[27]。

诊断ET时应重点强调与纤维化前/早期PMF（prefibrotic/early primary myelofibrosis，pre-PMF）的鉴别[1]–[2],[9],[13],[28]。一项纳入891例ET和180例pre-PMF患者的研究显示，pre-PMF患者的预后显著差于ET，10年总生存率分别为76％和89％，15年总生存率分别为59％和80％；10年进展为显著骨髓纤维化的比例分别为12.3％和0.8％，15年进展比例分别为16.9％和9.3％；10年进展为急性白血病的比例分别为5.8％和0.7％，15年进展比例分别为11.7％和2.1％[28]。骨髓病理评估对于鉴别ET和pre-PMF至关重要（表4）[1],[9]。

**表4:** 原发性血小板增多症与纤维化前/早期原发性骨髓纤维化病理鉴别要点

病理特点	原发性血小板增多症	纤维化前/早期原发性骨髓纤维化
骨髓增生程度	年龄校正后骨髓增生程度正常或轻度增高	年龄校正后骨髓增生程度显著增高
三系增生情况	粒系和红系增生正常且未见明显核左移；以巨核细胞增生为主	粒系显著增生，红系增生减低
巨核细胞分布	多呈散在或松散簇状分布（通常≤6个巨核细胞/簇）	常成簇分布（部分>6个巨核细胞/簇）
巨核细胞形态	细胞质和细胞核同步增大，细胞体积大至巨大，细胞核过分叶，呈“鹿角状”	细胞核体积增大程度超过细胞质，细胞体积大小不一，细胞核低分叶，呈“云朵状”

3. 遗传性血小板增多症：对于有家族史或血小板持续增多但无典型驱动基因突变的年轻患者，可进一步行靶向测序或全外显子组测序进行鉴别[23]–[25]。

4. 假性血小板增多症：外周血中小畸形红细胞、红细胞碎片或细胞质碎片可能被自动血细胞分析仪误判为血小板，见于地中海贫血、急性白血病、脓毒性休克等[14],[29]。此外，冷球蛋白在检测过程中可形成沉淀或小颗粒，同样可能被误识别为血小板，见于丙型肝炎、多发性骨髓瘤、淋巴瘤、间质性肾病或干燥综合征等[30]–[32]。镜下人工血小板计数可用于进一步确认是否存在假性血小板增多。采用激光散射法或荧光染色流式细胞计数法进行血小板测定，有助于区分血小板与细胞碎片或小红细胞，从而减少测定误差。

（四）预后判断标准

**临床问题4**：ET患者确诊后，应如何进行血栓风险评估？

**推荐意见4**：患者确诊后，推荐采用修订版原发性血小板增多症血栓风险国际预后评分系统（修订版IPSET-血栓）进行血栓危险度分层（强一致）[8]–[9],[33]–[34]。

推荐说明：修订版IPSET-血栓危险度分层：极低危（无血栓史、年龄≤60岁且JAK2 V617F阴性）；低危（无血栓史、年龄≤60岁且JAK2 V617F阳性）；中危（无血栓史、年龄>60岁且JAK2 V617F阴性）；高危（既往有血栓史；或年龄>60岁且JAK2 V617F阳性）[8]–[9],[33]–[34]。

对于合并CVRF的ET患者，可考虑将CVRF视为可能的附加血栓风险提示因素[34]–[35]。抗磷脂抗体阳性或抗磷脂综合征、蛋白C缺乏、蛋白S缺乏及血小板膜糖蛋白Ⅰa/Ⅱa c.807C>T多态性等，可能与MPN患者血栓风险增加相关，但目前证据仍有限[36]–[37]。对于合并上述因素的患者，临床实践中可考虑在修订版IPSET-血栓风险分层基础上，进行个体化、谨慎的风险评估（强一致）。

**临床问题5**：ET患者确诊后，应如何进行生存预后评估？

**推荐意见5.1**：推荐首选突变驱动的国际预后积分系统（MIPSS-ET）进行生存预后评估（表5）（强一致）[20]。

**表5:** 突变驱动的原发性血小板增多症国际预后积分系统（MIPSS-ET）

预后变量	积分
高风险基因突变（SRSF2突变、SF3B1突变、U2AF1突变或TP53突变）	2
年龄>60岁	4
男性	1
白细胞计数≥11×10^9^/L	1

**注** 风险组：低危：0～1分，中危：2～5分，高危：≥6分；低、中、高危组的中位生存期分别为34.4、14.1、7.9年

**推荐意见5.2**：对于无法开展二代测序的患者，建议采用MPN研究与治疗国际工作组（IWG-MRT）提出的ET国际预后积分系统（IPSET）（表6）（强一致）[38]。

**表6:** 骨髓增殖性肿瘤研究与治疗国际工作组原发性血小板增多症国际预后积分系统（IPSET）

预后变量	积分
年龄≥60岁	2
白细胞计数≥11×10^9^/L	1
既往血栓病史	1

**注** 风险组：低危：0分，中危：1～2分，高危：≥3分；低、中、高危组的中位生存期分别为未达到、24.5年、14.7年

（五）治疗

**临床问题6**：ET患者的治疗目标是什么？

**推荐意见6.1**：ET患者目前的主要治疗目标是预防和治疗血栓并发症（强一致）[8]–[9],[13]。

**推荐意见6.2**：建议高危ET患者的血液学治疗目标为达到完全血液学缓解（complete hematologic response，CHR）（PLT≤400×10^9^/L，WBC<10×10^9^/L，且外周血中无幼稚粒细胞、幼稚红细胞）（证据质量：低；条件推荐；强一致）[13],[39]–[41]。

推荐说明：一项来自西班牙的注册研究（按照修订版IPSET-血栓进行危险度分层）纳入1 080例ET患者，评估羟基脲治疗后达到CHR的预后价值。研究显示，高危组患者接受羟基脲治疗后达到CHR可显著降低动脉血栓风险（*HR*＝0.35，95％*CI*：0.20～0.60），同时呈现静脉血栓风险降低的趋势（*HR*＝0.45，95％*CI*：0.20～1.02）。此外，与未达缓解者相比，达到CHR的中危及高危组患者有更长的总体生存期，且骨髓纤维化进展风险更低[39]。

对于极低危、低危及中危组患者，当出现降细胞治疗指征或合并其他遗传性/获得性高凝状态时，应考虑结合个体血栓风险、出血风险及症状控制需求，制定个体化治疗目标。

**临床问题7**：不同风险分层的ET患者应遵循何种治疗原则？

**推荐意见7.1**：推荐基于修订版IPSET-血栓风险评分实施分层治疗：①极低危组（无血栓史、年龄≤60岁且JAK2 V617F阴性）：无微循环障碍症状者，观察随诊；出现微循环障碍症状者，予阿司匹林75～100 mg，每日1次。②低危组（无血栓史、年龄≤60岁且JAK2 V617F阳性）和中危组（无血栓史、年龄>60岁且JAK2 V617F阴性）：推荐予阿司匹林75～100 mg，每日1次。③高危组（既往有血栓史；或年龄>60岁且JAK2 V617F阳性）：推荐阿司匹林75～100 mg，每日1次联合降细胞治疗（强一致）[8]–[9],[11],[13],[42]。

推荐说明：极低危、低危和中危组患者如出现以下任一情况，应考虑启动降细胞治疗（强一致）[8]–[11],[13]：①新发血栓、获得性von Willebrand综合征（acquired von Willebrand syndrome，AVWS）和（或）与血小板增多相关的严重出血。②有症状的脾肿大或进行性脾肿大（需首先排除疾病进展）。③进行性血小板增多和（或）白细胞增多（需首先排除疾病进展）。④出现疾病相关症状（如瘙痒、夜间盗汗、乏力等），症状量化评估可采用MPN-SAF TSS：既往研究常将TSS>20分作为“具有临床意义的症状负担”的操作性阈值；单项症状评分4～6分提示中度、≥7分提示重度（上述阈值可供临床参考）[43]–[44]。⑤阿司匹林等抗血小板治疗无法控制的微循环障碍症状（如头痛、红斑性肢痛症等）。⑥低危组及中危组患者PLT>1 000×10^9^/L，或极低危组患者PLT>1 500×10^9^/L；对于极低危组患者，当PLT在（1 000～1 500）×10^9^/L范围内时，应综合个体血栓风险、出血风险及症状控制需求，制定个体化治疗策略。

**推荐意见7.2**：建议所有患者积极管理和控制CVRF（强一致）[8]。

**推荐意见7.3**：当PLT>1 000×10^9^/L或VWF水平<30％和（或）存在ET相关出血表现时，应慎用阿司匹林等抗血小板药物，可考虑通过降细胞治疗控制血小板水平或纠正VWF水平后再评估抗血小板治疗策略（强一致）[45]。

推荐说明：PLT明显升高（>1 000×10^9^/L）的患者可能存在AVWS或出血风险[45]。

**推荐意见7.4**：对于合并其他可能增加MPN血栓风险因素（如CVRF、抗磷脂抗体阳性/抗磷脂综合征、蛋白C缺乏、蛋白S缺乏等）的患者，应综合评估患者的血栓及出血风险，制定个体化的抗血小板、抗凝或降细胞治疗策略，必要时可参考《易栓症诊断与防治中国指南（2021年版）》及欧洲抗风湿病联盟成人抗磷脂综合征管理建议等相关指南（强一致）[37],[46]。

**推荐意见7.5**：进展为post-ET MF的患者，应参照骨髓纤维化的治疗原则进行管理；进展为急性白血病者，应参照MPN白血病期的治疗原则（强一致）[8]。

**临床问题8**：ET患者应如何选择抗血小板药物治疗？

**推荐意见8.1**：对于需接受抗血小板治疗的ET患者，推荐阿司匹林75～100 mg，每日1次（证据质量：低；强推荐；强一致）[8],[42]。

**推荐意见8.2**：如存在阿司匹林禁忌证或不耐受，可考虑使用氯吡格雷75 mg，每日1次（强一致）[8],[13]。

**推荐意见8.3**：对于接受阿司匹林75～100 mg每日1次治疗后仍存在ET相关症状（如肢端感觉异常、头痛等，应首先排除器质性病变）的患者，或仍发生动脉血栓事件者，可考虑更换为其他抗血小板药物（如氯吡格雷）进行单药治疗，或在特定情况下与阿司匹林联合应用。若上述策略仍不能有效控制症状，或患者存在明确的动脉血栓高风险，在充分评估出血风险后，可考虑将阿司匹林调整为75～100 mg，每日2次（证据质量：低；条件推荐；强一致）[8],[22],[47]。

推荐说明：其他抗血小板药物（如双嘧达莫、吲哚布芬、替格瑞洛等）在ET中尚缺乏有效性和安全性证据，仅在阿司匹林及氯吡格雷均不适用的少数患者或已使用以上药物且疗效和安全性明确的患者中，经充分评估血栓与出血风险后，谨慎且个体化地考虑替代方案（强一致）。

需要指出的是，大剂量阿司匹林（>100 mg/d）在ET患者中的有效性与安全性证据仍有限，尚需更多高质量研究加以验证[8],[22],[47]。

**临床问题9**：ET患者应如何选择降细胞治疗药物？

**推荐意见9.1**：推荐羟基脲或干扰素α制剂为ET患者的一线降细胞药物（证据质量：高；强推荐；强一致）；对于年龄<60岁的年轻患者，推荐首选干扰素α制剂（证据质量：高；强推荐；强一致）[8]–[9],[11],[22]。

推荐说明：多项研究已证实，羟基脲能够降低高危ET患者的血栓风险[48]–[49]。推荐起始剂量为15～20 mg·kg^−1^·d^−1^，1周后根据血液学指标逐渐调整剂量，直至达到理想疗效后长期维持（证据质量：高；强推荐）[9],[13],[22]。

SURPASS-ET研究表明，罗培干扰素α-2b（Ropeg-IFNα-2b）在羟基脲耐药/不耐受ET患者中的血液学缓解率、分子学缓解率及安全性均优于阿那格雷[50]。Ropeg-IFNα-2b推荐剂量：起始剂量为250 µg皮下注射，2周后加量至350 µg，再经2周加量至目标剂量500 µg，每2周1次，用药期间根据血液学指标调整剂量，直至达到理想疗效后长期维持（证据质量：高；强推荐）[9],[50]。

MPD-RC112研究表明，聚乙二醇干扰素α（Peg-IFNα）在高危ET患者中的长期血液学缓解率及分子学缓解率均优于羟基脲，但3/4级不良事件发生率较高[17]。Peg-IFNα-2a/2b推荐剂量：起始剂量为90～135 µg皮下注射，每周1次，1～2周后依据不良反应和疗效逐渐增加剂量至135～180 µg，每周1次。随后根据血液学指标调整剂量，直至达到理想疗效后长期维持（证据质量：高；强推荐）[9],[17]。

普通剂型干扰素α：起始剂量为900～2 500万单位/周，分3次皮下注射，治疗1～2周后根据血液学指标逐渐调整剂量，直至达到理想疗效后长期维持[13]。

**推荐意见9.2**：对于需要二线降细胞治疗的患者，可考虑使用阿那格雷（证据质量：中；条件推荐；强一致）[13],[49],[51]。

推荐说明：多项随机对照研究显示，阿那格雷在高危ET患者中的血液学疗效与羟基脲相近，但在血栓、出血及骨髓纤维化转化风险方面，不同研究的结果尚不一致[49],[51]–[52]。推荐剂量：阿那格雷起始剂量0.5 mg每日2次；或1 mg每日1次。最大单次剂量2.5 mg，每日最大剂量为10 mg，至少1周后再开始剂量调整，每周剂量增加不超过0.5 mg/d（证据质量：中；条件推荐）[13],[49],[51]。

**推荐意见9.3**：对上述降细胞治疗药物均耐药或不耐受的部分患者，可考虑JAK1/2抑制剂芦可替尼（证据质量：中；条件推荐；强一致）[8],[53]。

推荐说明：在MAJIC-ET研究中，芦可替尼与最佳可用治疗（best available therapy，BAT）的CHR率相当，在改善ET相关症状方面优于BAT，但3/4级贫血及血小板减少的发生率较高[53]。芦可替尼推荐剂量：起始剂量为10 mg，每日2次，随后根据血液学指标逐渐调整剂量，直至达到理想疗效后长期维持（证据质量：中；条件推荐）[8],[53]。

**推荐意见9.4**：使用降细胞治疗的患者需动态监测治疗反应，如出现耐药或不耐受、新发血栓、AVWS和（或）与血小板增多相关的严重出血、脾脏进行性增大、进行性白细胞和（或）血小板增多、疾病相关症状不能缓解或加重，或微循环障碍症状控制不佳等情况时，应考虑调整治疗方案（强一致）[8]–[9],[11],[13]。

推荐说明：使用降细胞治疗的患者如出现以下任一新发情况，应考虑调整治疗方案：①对初始降细胞治疗出现耐药或不耐受；②出现新发血栓、AVWS和（或）与血小板增多相关的严重出血；③脾脏进行性增大（需首先排除疾病进展）；④进行性血小板增多和（或）白细胞增多（需首先排除疾病进展）；⑤疾病相关症状（如瘙痒、夜间盗汗、乏力等）不能缓解或加重；⑥阿司匹林等抗血小板治疗无法控制的微循环障碍症状（如头痛、红斑性肢痛症等）[8]–[9],[11],[13]。

治疗方案首选参加临床试验或更换为既往未使用过的降细胞药物（羟基脲与干扰素α制剂可互为一线治疗失败后的二线选择；当羟基脲和干扰素α均治疗失败时，可考虑使用阿那格雷），必要时酌情联合使用以上药物。对于上述药物控制不佳的患者，部分情况下可考虑使用芦可替尼（强一致）[8],[44]。

**推荐意见9.5**：对于出现严重神经系统并发症、危及生命的急性血栓事件或严重出血事件，伴PLT明显升高（>1 000×10⁹/L）且难以控制者，可考虑进行血小板单采治疗（强一致）[8],[41]。

推荐说明（研究进展）：目前，针对ET的多项新型治疗策略正在开展临床研究，具有潜在的治疗价值，包括赖氨酸特异性组蛋白去甲基化酶1抑制剂bomedemstat、选择性JAK2抑制剂OB756及靶向CALR突变体的单克隆抗体INCA033989等[54]–[56]。此外，靶向CALR突变体的T细胞重定向双特异性抗体、抗体-药物偶联物及CAR-T细胞疗法尚处于临床前研究阶段[57]。

ET降细胞治疗给药方案及注意事项见表7。

**表7:** 原发性血小板增多症降细胞治疗给药方案及注意事项

药物	起始剂量	给药方式	注意事项
一线药物			
羟基脲	15～20 mg·kg^−1^·d^−1^	口服	常见不良反应包括骨髓抑制、低热、胃肠道不适及皮肤/黏膜反应（干燥、皮疹、口腔黏膜溃疡、皮肤溃疡）等，亦可见指（趾）甲色素沉着，少数患者可出现肝功能异常（如转氨酶升高）或脱发
罗培干扰素α-2b	250 µg，2周后加量至350 µg，再经2周加量至500 µg，每2周1次	皮下注射	干扰素α制剂的常见不良反应包括流感样症状、皮疹、乏力、脱发、食欲不振、肝功能异常、甲状腺功能异常，伴自身免疫性疾病、视网膜病变及抑郁等精神症状，使用前应完善甲状腺功能、肝功能、风湿免疫及眼科检查，并询问是否有精神疾病史，治疗过程中密切监测上述指标及临床表现
聚乙二醇干扰素α-2a/2b	90～135 µg，每周1次，1～2周后依据不良反应和疗效增加至135～180 µg，每周1次	皮下注射	
普通剂型干扰素α	900～2 500万单位/周，分3次	皮下注射	
二线药物			
阿那格雷	0.5 mg每日2次；或1 mg每日1次	口服	可能引起心悸、心律失常、体液潴留、心力衰竭、头痛或头晕等不良反应，老年患者及有心脏基础疾病者应慎用
其他药物			
芦可替尼	10 mg，每日2次	口服	停药/中断可能出现撤药综合征（发热、呼吸窘迫、低血压、多器官衰竭等）；除血小板或中性粒细胞明显减少等原因外，不建议骤停，应考虑逐渐减量；必要时可考虑糖皮质激素用于撤药综合征

成人ET的诊治流程见图1。

**图1:**
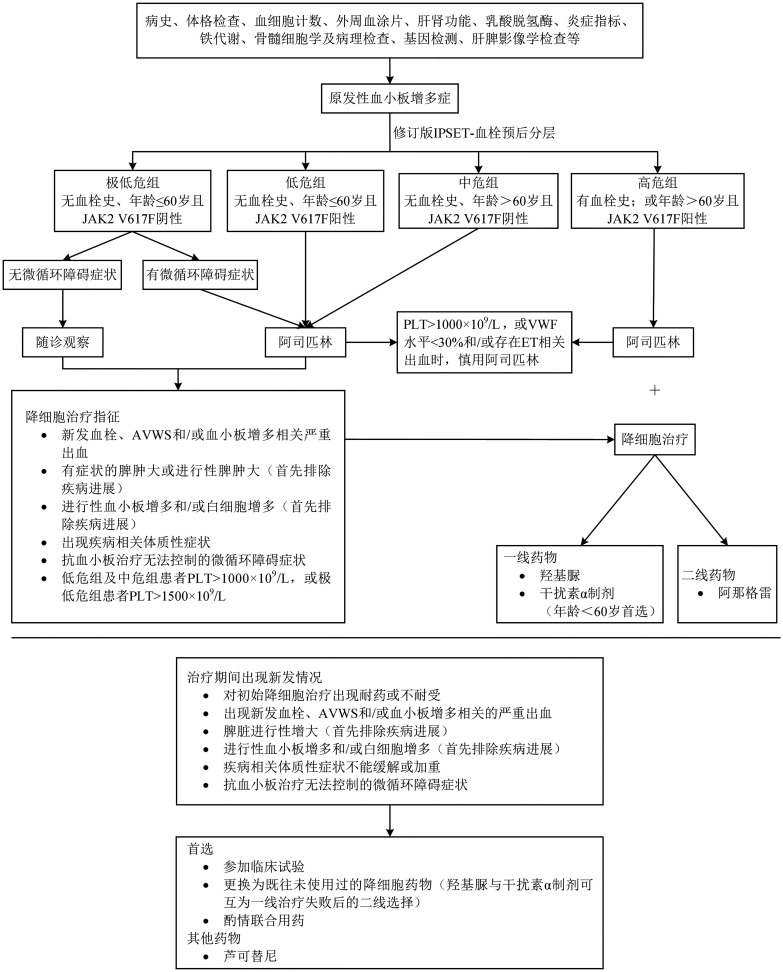
成人原发性血小板增多症（ET）诊治流程 **注** IPSET：原发性血小板增多症国际预后评分系统；VWF：血管性血友病因子；AVWS：获得性von Willebrand综合征

**临床问题10**：ET患者发生动脉血栓事件后应如何处理？

**推荐意见10.1**：对于发生血栓事件的ET患者，推荐予规范的降细胞治疗（强一致）[11]。

**推荐意见10.2**：对于既往未使用抗血小板药物而发生动脉血栓的患者，应根据血栓部位参照相应专科指南及时启动抗血小板治疗或血管再通治疗。动脉血栓事件稳定后，推荐长期（终生）予小剂量阿司匹林75～100 mg，每日1次作为二级预防，并严格管理CVRF（强一致）[58]。

**推荐意见10.3**：若动脉血栓发生于患者已因既往动脉血栓接受抗血小板治疗期间，建议多学科会诊，根据血栓部位、合并症及出血风险选择最适宜的个体化抗血栓方案。可由相关团队评估是否需介入或血管再通治疗，并根据具体情况考虑更换抗血小板药物、酌情采用双联抗血小板治疗、调整阿司匹林剂量，或评估直接口服抗凝药（direct oral anticoagulants，DOAC）的应用（强一致）[59]。

**临床问题11**：ET患者发生静脉血栓事件后应如何处理？

**推荐意见11.1**：ET患者发生静脉血栓栓塞症（venous thromboembolism，VTE，包括深静脉血栓和肺栓塞）时，其初始治疗原则总体与普通人群一致（强一致）[8]。抗凝治疗建议以低分子肝素（low-molecular-weight heparin，LMWH）作为起始方案，病情稳定后，可依据血栓部位相关专科指南，过渡至维生素K拮抗剂（vitamin K antagonist，VKA）或DOAC进行维持治疗（证据质量：低；条件推荐；强一致）[60]–[63]。

推荐说明：目前，DOAC在ET患者中的应用证据主要来源于观察性研究，疗效与安全性仍需更多大样本前瞻性研究进一步验证，建议结合血栓部位、合并症及出血风险等因素进行个体化药物选择[62],[64]–[65]。抗凝治疗联合阿司匹林可能较单用阿司匹林增加出血风险[66]。阿司匹林与抗凝治疗的联合应用应在充分评估获益与风险后个体化决策。

**推荐意见11.2**：抗凝治疗的持续时间应根据血栓事件的严重程度、ET疾病控制情况及停用抗凝治疗后血栓复发风险的综合评估来确定（强一致）[59],[67]。

推荐说明：对于复发风险较高或存在高危VTE的患者，如对降细胞治疗反应欠佳、复发性VTE、内脏静脉血栓形成、危及生命的VTE及伴有先天性或获得性高凝状态，可考虑延长抗凝治疗时间[10],[68]。在停止抗凝治疗后，可考虑恢复阿司匹林治疗作为持续的抗血小板预防措施[67]。抗凝治疗期间需定期监测血常规及肝肾功能，并动态评估再发血栓与出血风险，以便及时调整治疗策略。

对于反复血栓或出现严重血栓事件的患者，如相关检测可能影响抗凝强度或治疗时长，应考虑筛查遗传性和获得性高凝状态[8]。相关评估可参考《易栓症诊断与防治中国指南（2021年版）》[37]。

**推荐意见11.3**：对于部分严重或特殊部位静脉血栓事件患者，建议由相关专科或多学科团队评估是否需要进一步介入治疗（强一致）[8]。

**临床问题12**：妊娠期ET患者应如何进行管理和治疗？

**推荐意见12.1**：建议进行孕前会诊，并由血液科与产科医师在备孕及整个妊娠期间共同制定和实施管理方案。计划妊娠前3个月（不论男女）或确诊妊娠后应立即停用羟基脲或阿那格雷（强一致）[8]–[9],[13],[69]–[72]。

**推荐意见12.2**：对于无妊娠高危因素的ET患者，如无禁忌证，推荐妊娠期间全程使用低剂量阿司匹林75～100 mg，每日1次，并在产后6周内单用预防剂量LMWH（证据质量：低；强推荐；强一致）[8]–[9],[13],[72]–[74]。

**推荐意见12.3**：对于具有妊娠高危因素的ET患者，在无明确禁忌的前提下，推荐妊娠期间使用阿司匹林75～100 mg，每日1次联合预防剂量LMWH；LMWH持续至产后6周（证据质量：低；强推荐；强一致）[8]–[9],[13],[72]–[74]。

推荐说明：ET患者在妊娠期间面临流产、早产、胎儿宫内生长受限、母体血栓或出血及母体先兆子痫等风险[69]–[70]。

ET女性患者高危妊娠的定义（符合以下任意一项）：既往有动脉或静脉血栓史（无论是否妊娠）；既往有ET相关出血史（无论是否妊娠）；既往发生过以下可能由ET引起的妊娠并发症：反复（≥3次）发生不明原因所致妊娠10周内流产（无胎儿或胎盘结构、性激素或染色体异常）；不能解释的胎儿宫内生长受限；妊娠≥10周，发育正常胎儿不明原因宫内死亡；因严重先兆子痫、子痫或胎盘功能不全导致的<34周且胎儿发育正常的早产、严重的产前和产后出血（需红细胞输注）[8]–[9],[13],[72]。

若患者在妊娠前已使用低剂量阿司匹林，可在完成产后LMWH疗程后恢复阿司匹林治疗。对于孕前因静脉和（或）动脉血栓事件接受抗凝治疗的ET患者，妊娠期间应继续使用LMWH（治疗剂量或按风险个体化剂量），结合血栓风险、合并症及出血风险进行剂量调整及评估产后抗凝治疗的持续时间[8],[13],[72]–[74]。

一般情况下，预计分娩前2周建议停用阿司匹林，并以LMWH替代，LMWH应于分娩前12～24 h停用。分娩前停用LMWH和阿司匹林的具体时机及产后重新开始用药的时机应由血液科医师、产科医师及产科麻醉医师共同评估决定[8]。对于既往有ET相关出血史的患者，应避免LMWH预防性治疗[8]。

**推荐意见12.4**：合并妊娠高危因素或血小板极度增多的ET患者，可作为妊娠期降细胞治疗的潜在适应人群（强一致）[8]–[9],[13],[72],[75]。

推荐说明：既往部分国内外指南/共识提出，当PLT>1 500×10⁹/L时可考虑启动降细胞治疗，但相关依据主要来自共识性意见，临床可作为参考（强一致）[8]–[9],[13],[72],[75]。

**推荐意见12.5**：妊娠期应避免使用羟基脲或阿那格雷，如需降细胞治疗，可考虑使用干扰素α制剂（证据质量：低；条件推荐；强一致）[8],[13],[72],[76]–[78]。

推荐说明：若孕早期发生无意羟基脲暴露，应由血液科与产科医师共同评估暴露风险，综合决定是否继续妊娠，并根据病情需要考虑是否更换为干扰素α制剂[78]。

一篇系统性综述纳入22项研究、共767例MPN女性患者，涉及1 210次妊娠，ET患者的总体活产率为71.1％。无论是单用干扰素α，还是联合阿司匹林或肝素治疗，均与更高的活产率相关，各治疗方案的母体并发症发生率无显著差异[77]。另一项系统性综述（纳入504例ET女性，涉及756次妊娠）显示，总体活产率为74％。使用LMWH者母体VTE风险较未使用LMWH者更低（0对2.5％），阿司匹林对母体VTE风险无显著影响，而干扰素α治疗组的母体VTE风险较未使用干扰素α治疗组更低（0对2％）。在产褥期，LMWH同样可降低母体VTE风险（0对4.4％），且未增加出血风险[74]。需要指出的是，妊娠期使用干扰素α制剂的有效性及安全性证据主要来自回顾性研究及系统性综述，临床应用时应在预期获益明确高于对母体与胎儿潜在风险的前提下慎重选择，治疗前与患者充分沟通并评估禁忌证，治疗过程中加强母体与胎儿的不良反应监测。

妊娠期间建议定期监测血常规、体重、肝肾功能及胎儿生长发育情况，可于孕20周左右行子宫动脉多普勒检查，同时动态评估血栓与出血风险，及时个体化调整抗血小板、抗凝及降细胞治疗方案[8],[79]。对于因反复血栓形成而流产的患者，应考虑进一步筛查其他可能导致血栓的原因，包括遗传性及获得性高凝状态等[8],[37]。

低剂量阿司匹林仅有少量分泌入乳汁，LMWH不经乳汁排泄，二者均不影响哺乳。干扰素α制剂在乳汁中含量极低，且口服吸收度差，但目前尚缺乏安全性数据[80]。哺乳期女性应避免使用羟基脲、阿那格雷或DOAC[8]–[9],[13]。

**推荐意见12.6**：当血小板显著增多（PLT>1 000×10^9^/L）或合并AVWS和（或）ET相关出血表现时，应综合评估血栓与出血风险，个体化制定抗血小板、抗凝及降细胞治疗策略（强一致）[45],[79]。

推荐说明：既往研究显示，ET合并AVWS时，VWF异常在妊娠过程中可能随孕周推进而改善，且母体出血事件及妊娠结局总体较好。该研究提出，拟妊娠的ET患者可于孕前检测凝血因子Ⅷ活性及VWF水平，如存在AVWS或ET相关出血表现，动态复查相关指标以辅助用药决策，并于妊娠第三孕期复查以指导分娩管理。该研究为ET合并AVWS妊娠患者的管理及临床结局提供了参考，但仍需进一步研究以明确最佳监测与管理策略[79]。

（六）疗效判断

**临床问题13**：ET患者应采用何种标准进行疗效评价？

**推荐意见13**：推荐采用欧洲白血病网和IWG-MRT于2013年修订的ET疗效评价标准（表8）（强一致）[81]。

**表8:** 原发性血小板增多症（ET）的疗效判断标准

疗效标准	定义
完全缓解	以下4条必须全部符合： ①可触及的肝脾肿大等疾病相关体征持续（≥12周）消失，症状显著改善（MPN-SAF TSS评分下降≥10分）； ②外周血细胞计数持续（≥12周）缓解：PLT≤400×10^9^/L，WBC<10×10^9^/L，外周血无幼稚粒细胞、幼稚红细胞； ③无疾病进展，无任何出血或血栓事件； ④骨髓组织学缓解：巨核细胞显著增生消失，无>1级的网状纤维（欧洲分级标准）
部分缓解	以下4条必须全部符合： ①可触及的肝脾肿大等疾病相关体征持续（≥12周）消失，症状显著改善（MPN-SAF TSS评分下降≥10分）； ②外周血细胞计数持续（≥12周）缓解：PLT≤400×10^9^/L，WBC<10×10^9^/L，外周血无幼稚粒细胞、幼稚红细胞； ③无疾病进展，无任何出血或血栓事件； ④无骨髓组织学缓解：巨核细胞显著增生
无效	疗效未达到部分缓解
疾病进展	进展为真性红细胞增多症、ET后骨髓纤维化、骨髓增生异常肿瘤或急性白血病

**注** MPN-SAF TSS：骨髓增殖性肿瘤总症状评估量表

推荐说明：分子生物学疗效对于评价完全缓解或部分缓解不是必需的。完全分子生物学缓解：原先存在的分子异常完全消失。部分分子生物学缓解：基线等位基因突变负荷≥20％的患者在治疗后等位基因突变负荷较基线下降≥50％（强一致）[13],[81]。

**临床问题14**：ET患者羟基脲耐药或不耐受应采用何种判断标准？

**推荐意见14**：推荐采用2011年欧洲白血病网标准判断ET患者羟基脲耐药或不耐受（表9）（强一致）[82]。

**表9:** 原发性血小板增多症患者羟基脲耐药或不耐受标准

羟基脲耐药或不耐受标准（符合以下任意一项）
1. 在羟基脲剂量≥2.0 g/d（体重>80 kg者为≥2.5 g/d）并治疗≥3个月后，血小板计数仍>600×10⁹/L；
2. 在任意剂量羟基脲治疗期间，血小板计数>400×10^9^/L，且白细胞计数<2.5×10^9^/L；
3. 在任意剂量羟基脲治疗期间，血小板计数>400×10^9^/L，且血红蛋白<100 g/L；
4. 在羟基脲治疗过程中出现下肢溃疡，或其他不能耐受的皮肤或黏膜不良反应；
5. 出现与羟基脲相关的持续性发热
